# Natural History of the Fœtid Gums

**Published:** 1809-07

**Authors:** 


### Natural History of the fœtid Gums.

( Continued from our last. )

BUBON GALBANUM, is another plant of the natural order of umbellatæ, and stands, according to the Linnæan System, in the same class and order as the preceding. The different colleges in their pharmacopœia have designated the Bubon Galbanum as shrubby, with compound leaves issuing from the sheaths of the stem, tripinnated, veined, and of a sea green colour. The principal umbell terminates the stem, where it radiates with some regularity. The flowers are all hermaphrodite, and produce prolific seeds.

In this, as in the former instance, however, we are not perfectly certain of the plant. From the diversity of authors, some of them must be mistaken, or Galbanum must be procured from more than one species of Bubon. The latter is more consistent with candour as well as probability. The drug, as we see it, is usually imported to us from Turkey. It is a tenacious dry gum resin, white in proportion as it is fresh, of a smell generally not grateful, of a pungent bitter taste. It transudes spontaneously, but more readily from incisions in the joints of plants of 3 or 4 years growth. But it is said the best way is to cut the trunk transversely about 3 or 4 fingers above its root, from which the gum will fall in drops. These drops, or distinct grains, are by Mr. Geoffrey called tears, in distinction to larger masses, which he calls loaves. That in tears is esteemed the purest, but the masses are easily detected by numerous white speculæ, which sometimes appear to shine, and which tinge spirit of wine with a golden hue. The worst kind is of a dusky brown colour, and of the consistence of wax. The smell to those who possess this sense with sufficient accuracy, will always betray any sophistication of this article. When exposed to the flame of a candle, it burns and slightly deflagrates, with a white flame and copious smoke, which has an aromatic smell, but a considerable part is left unconsumed. It is dissolved in either water or spirit of wine, but not in oils. In a menstruum of two parts of spirit and one of water, it may be perfectly dissolved: by which it appears that it contains double the quantity of resin to that of gum. Rubbed with water, it becomes milky, but precipitates afterwards.

By distillation, we are informed by Newman, it yields an essential oil, which swims in water; if distilled per se, it affords a strong empyreumatic oil.

The virtues of Galbanum are very considerable, though not equal to assafœtida. It is, however, less disgusting in its smell, and therefore of more general use. As a pectoral, it is less esteemed than ammoniacum. Externally applied in the old *emplaste gummis,* it is particularly convenient for those boils, which from their situation, do not conveniently admit poultices, and as long as the cuticle is preserved, its application will be found serviceable in whatever may be the stage of suppuration.

Bubon Macedonicum, formerly called Petroselinum Macedonicum. Since the omission of the larger composition of Theriaca and Mithridate, has not found a place in the London Pharmacopœia, consequently as its virtues could not be well ascertained among so many more powerful remedies, may be omitted as to any other notice than a mere demonstration in cabinets.

Gummi Sagapenum.—Though this gum has been long in use, and was even mentioned, as we have reason to believe, by Diascorides; yet we are to this day ignorant of the plant that produces it. It appears, however, by the specimens brought over, which contain portions of, and sometimes whole seeds, to be an umbellated plant, and as it it has something of the smell of galbanum and assafœtida, probably is of a species not very far removed from the latter.

The specimens usually contain masses consisting of parts of very different consistence, so that there is reason to believe the whole made up of tears, as they are usually called, transuded at different times from some part of the plant. Sometimes, indeed, we meet with small clean pieces, which have not the appearance of fragments, but these are, I believe, at present, very uncommon.

Sagapenum yields much of its smell and flavour to the still, affording an essential oil and water highly impregnated. About three-fourths of the gum-resin are resolved by long boiling in water, into a turbid yellowish white liquor. Rectified spirits will take up about half, and is very little coloured. The solution smells very weak, and tastes very strong. By this, it is probable that the more volatile parts are dissolved in the watery menstruum.

Sagapenum, as its external qualities show, has many of the virtues both of assafœtida and Galbanum. It has the advantage of both, in proving for the most part somewhat cathartic, a consideration deserving attention, inasmuch as hysterical and hypochondriacal subjects have usually a great torpidity in this and most other secretions, excepting the urinary. On this account, it was formerly more used for what is called resolving obstructions in the different viscera, hypochondria, spleen, and mesentery.

Tacamahac; this gum-resin has generally been supposed to be procured from the populus *Balsamifera* of Linnæus. In this way it is described by Lewis, and in the early volumes of Murray, who commends it as an antirheumatic. But Bergius assures us that no populus yields a substance at all resembling Tacamahac; and Murray in his last volume, published in 1792, admits the justice of the remark. He goes further, and acknowledges his mistake in the virtues he at one time imputed to this drug, assuring us, that used by fumigation as was directed, it is either altogether useless or injurious, driving the peccant matter of rheumatism to the bones, and still more important organs. However this may be, there is reason to suspect that a remedy of real value would have been more used, or at least, that its virtues would have been by this time well ascertained.

